# Ischemic stroke after COVID-19 bivalent vaccine administration in patients aged 65 years and older in the United States

**DOI:** 10.1038/s41541-023-00777-w

**Published:** 2023-11-23

**Authors:** Maria P. Gorenflo, Pamela B. Davis, David C. Kaelber, Rong Xu

**Affiliations:** 1 Center for Artificial Intelligence in Drug Discovery, Case Western Reserve University School of Medicine, Cleveland, OH USA; 2 Center for Community Health Integration, Case Western Reserve University School of Medicine, Cleveland, OH USA; 3 Center for Clinical Informatics Research and Education, The MetroHealth System, and Department of Internal Medicine, Pediatrics, and Population and Quantitative Health Sciences, Case Western Reserve University, Cleveland, OH USA

**Keywords:** Risk factors, Outcomes research

## Abstract

The Centers for Disease Control and Prevention announced in January 2023 a potential connection between administration of the Pfizer novel coronavirus disease-2019 (COVID-19) bivalent vaccine booster and ischemic stroke (IS). A retrospective cohort study was conducted to compare the hazard of IS in patients aged 65 years and over administered the Pfizer bivalent booster versus those administered the Pfizer/Moderna monovalent or Moderna bivalent boosters. De-identified patient electronic health data were collected from TriNetX, a cloud-based analytics platform that includes data from over 90 million unique patients in the United States. Patients aged 65 years and over at the time of administration of a Pfizer bivalent, Moderna bivalent, or Pfizer/Moderna monovalent booster were included for analysis. Cohorts were propensity-score matched. The hazard ratios (HR) and 95% confidence intervals (CI) for IS between matched cohorts at 1–21 and 22–42 days after booster administration were calculated. There was reduced hazard of IS in the Pfizer bivalent cohort compared to the monovalent cohort at both timepoints: 1–21 days after vaccination (HR: 0.54, 95% CI: 0.47–0.62), and 22–42 days after vaccination (HR: 0.62, 95% CI: 0.54–0.72) (*n* = 79,036 patients per cohort). There was reduced hazard of IS in the Pfizer bivalent cohort compared to the Moderna bivalent cohort at 1–21 days after vaccination (HR: 0.75, 95% CI: 0.58–0.96) (*n* = 26,962 patients per cohort). This analysis provides no evidence that the Pfizer bivalent vaccine is associated with increased hazard of IS compared to the monovalent or Moderna bivalent vaccines.

## Introduction

In January 2023, the Centers for Disease Control and Prevention (CDC) announced that their Vaccine Safety Datalink met the threshold to investigate the risk of ischemic stroke (IS) within three weeks of administration of the Pfizer/BioNTech novel coronavirus disease-2019 (COVID-19) bivalent vaccine (Pfizer bivalent booster)^1^. No such concern was raised in the CDC’s statement for the Moderna COVID-19 bivalent vaccine (Moderna bivalent booster). The earlier monovalent Moderna and Pfizer vaccines display no increased risk for IS in the general American population^2^; however, COVID-19 infection itself appears to be a risk factor for IS in patients ages 65 years and over^3^. This CDC announcement was therefore unanticipated, and since then both the Food and Drug Administration and European Medicines Agency have reported no increased IS risk for the Pfizer bivalent vaccine in their respective databases^4,5^. A recent analysis from the French National Health Data System has come to similar conclusions^6^. In response to these inconsistent findings and the wide use of COVID-19 bivalent vaccines in older adults in the United States, we set out to examine the comparative hazard of IS in patients ages 65 years and over who were administered the Pfizer bivalent booster, Moderna bivalent booster, or Pfizer/Moderna monovalent booster.

## Results

The study population of patients aged 65 years and over at the time of booster administration on or before August 27, 2023, included 110,667 who received the Pfizer bivalent booster, 26,962 who received the Moderna bivalent booster, and 96,156 who received a monovalent booster. Most monovalent vaccine booster doses were administered between August 2021 and February 2022, while most bivalent vaccine booster doses were administered between September 2022 and May 2023. The Pfizer bivalent booster cohort did not differ significantly from the monovalent cohort at baseline in demographics and had a significantly higher prevalence of pre-existing medical conditions, including COVID-19 (Table 1). After matching for the primary analysis, the two cohorts were balanced, and there were 79,036 patients in each cohort (Table 1). Details on the propensity-score matching results between the Pfizer bivalent and Moderna bivalent cohorts are available in Supplementary Table 1; for this analysis, there were 26,962 patients in each cohort after matching.

**Table 1:** Patient characteristics in the Pfizer bivalent cohort and monovalent cohort before and after propensity-score matching.

	Before matching, No. (%)			After matching, No. (%)		
	Pfizer bivalent cohort (*n* = 110,667)	Monovalent cohort (*n* = 96,156)	Standard Mean Difference	Pfizer bivalent cohort (*n* = 79,036)	Monovalent cohort (*n* = 79,036)	Standard Mean Difference
Age at Index (years, mean ± SD)	73.47 ± 6.15	73.65 ± 5.81	0.03	73.63 ± 6.08	73.61 ± 5.78	<0.01
Sex (%)						
Male	42.08	45.13	0.06	44.33	44.62	0.01
Female	52.69	54.55	0.04	55.21	54.98	<0.01
Race (%)						
White	70.89	72.52	0.04	74.73	75.3	0.01
Black or African American	12.18	13.47	0.04	12.77	12.76	<0.01
Asian	4.08	7.49	0.15^a^	5.18	5.01	0.01
Unknown Race	12.09	6.22	0.20^a^	6.96	6.56	0.02
Ethnicity (%)	6.75	9.61	0.10^a^	7.89	7.83	<0.01
Hispanic or Latino	79.13	86.11	0.19^a^	86.9	86.96	<0.01
Not Hispanic or Latino	14.13	4.28	0.35^a^	5.21	5.21	<0.01
Unknown Ethnicity	70.89	72.52	0.04	44.33	44.62	<0.01
Medical conditions (%)						
Disorders of lipoprotein metabolism and other lipidemias	73.83	60.2	0.29^a^	69.93	70.32	0.01
Essential (primary) hypertension	70.93	60.79	0.22^a^	67.89	67.95	<0.01
Type 2 diabetes mellitus	32.05	24.5	0.17^a^	27.85	28.11	0.01
Ischemic heart diseases	28.05	25.77	0.05	27.17	27.85	0.02
Overweight and obesity	32.42	26.02	0.14^a^	29.37	29.54	<0.01
Cerebrovascular diseases	17.03	14	0.08	15.33	15.7	0.01
Mental and behavioral disorders due to psychoactive substance use	16.73	10.25	0.19^a^	12.2	12.28	<0.01
Nicotine dependence	11.95	7.62	0.15^a^	9.06	9.09	<0.01
Atrial fibrillation and flutter	13.62	11.77	0.06	12.67	13.01	0.01
Cerebral infarction	6.28	5.02	0.05	5.54	5.68	0.01
COVID-19	13.86	4.89	0.31^a^	6.03	5.95	<0.01
Alcohol-related disorders	4.5	2.48	0.11^a^	2.94	3	<0.01
Persons with potential health hazards related to socioeconomic and psychosocial circumstances (%)	7.1	2.42	0.22^a^	3.06	2.94	0.01

^a^Standard mean difference greater than 0.1, a threshold indicating imbalance.

**Fig. 1:**
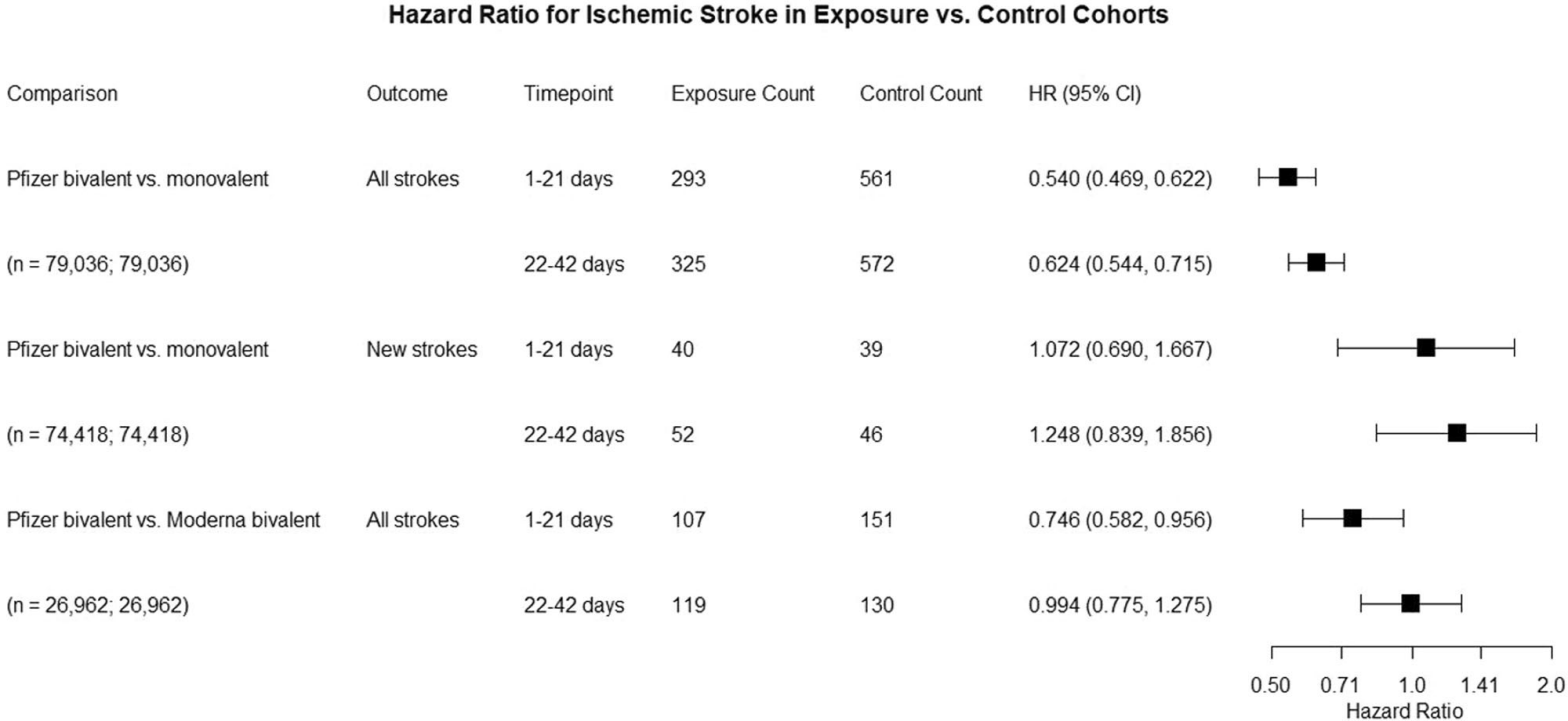
Risk of ischemic stroke following COVID-19 vaccine administration. Comparison of ischemic stroke hazard in the 1–21- and 22–42-day time-windows that followed from the day of vaccine administration between propensity-score matched Pfizer bivalent and monovalent cohorts (all strokes, first-time [“new”] strokes), and between propensity-score matched Pfizer bivalent and Moderna bivalent cohorts (all strokes).

There was reduced hazard of an IS encounter diagnosis in the Pfizer bivalent cohort compared to the matched monovalent cohort at 1–21- and 22–42-days post-administration: Hazard Ratio (HR) = 0.54, 95% Confidence Interval (CI) (0.47–0.62), and HR = 0.62, 95% CI (0.54–0.72), respectively (Fig. 1). There was reduced hazard of an IS encounter diagnosis in the Pfizer bivalent cohort compared to the matched Moderna bivalent cohort at 1-21 days: HR = 0.75, 95% CI (0.58–0.96), and no difference was observed at 22–42 days post-administration: HR = 0.99, 95% CI (0.78–1.28) (Fig. 1). There was no difference in the hazard of first-time IS encounter diagnoses between the Pfizer bivalent cohort and the matched monovalent cohort at either timepoint: HR = 1.07, 95% CI (0.69–1.67), and HR = 1.25, 95% CI (0.84, 1.86), respectively (Fig. 1).

## Discussion

We observed a reduced hazard of IS encounter diagnosis in the Pfizer bivalent cohort compared to the monovalent cohort, perhaps due to bivalent boosters providing stronger protection against severe COVID-19 infection and hospitalization than their monovalent counterparts^7,8^. Additionally, severe COVID-19 infection increases the risk of IS^9^, and the Omicron strain (dominant in 2022 when bivalent vaccines were distributed) produces less severe disease than the Delta strain (dominant in 2021 when monovalent vaccines were distributed)^10^, so perhaps this explains why patients administered the Pfizer bivalent booster displayed reduced hazard of an IS encounter diagnosis. There was also significantly reduced hazard of IS encounter diagnosis in the Pfizer bivalent versus Moderna bivalent cohorts, but only at the 1–21 day period. Additionally, there was no significant difference in the hazard of first-time IS encounter diagnosis between patients in the Pfizer bivalent and monovalent cohorts. Patients with prior stroke are at high risk for subsequent stroke due to inflammatory and vascular factors^11^ that severe COVID-19 may further exacerbate, whereas patients without prior stroke lack these factors.

There are case reports of IS being associated with vaccine-induced immune thrombotic thrombocytopenia^12^, so this may explain why some patients in our cohorts experienced IS after vaccination. Considering the high incidence of IS in the general population (as the fifth leading cause of death in the United States) and the high prevalence of IS risk factors in both cohorts (older age, dyslipidemia, hypertension, type II diabetes mellitus, overweight/obesity, and cerebrovascular disease amongst others)^13^, it is likely that a greater etiology of these strokes are the classic risk factors that were going to cause them regardless of COVID-19 vaccination. The CDC warned of a potentially increased risk of IS within three weeks of administration of Pfizer bivalent boosters^1^; this may be a reflection of the higher prevalence of pre-existing medical conditions that are IS risk factors among patients who received the Pfizer bivalent booster compared to those who received the monovalent booster (Table 1).

Limitations of this study include the use of the TriNetX platform, which is not a random sampling of the entire United States population over the age of 65 years; therefore, the generalizability of these results needs to be tested in other cohorts. Both Pfizer and Moderna bivalent vaccines were approved in August 2022; however, monovalent vaccines were approved earlier. While patients in the Pfizer bivalent cohort and the monovalent cohort were followed for the same length of time, the dominant SARS-Cov-2 variants that patients in these two cohorts encountered were different, which may confound the results. Additionally, this study does not include a complete sample of those who were vaccinated in the population of interest because many vaccines were administered outside of the healthcare organizations (HCOs) that report data to the TriNetX platform. In this study, patients were followed for up to six weeks; future studies should examine longer-term associations between COVID-19 vaccination and IS. In summary, our analysis provides no evidence that American patients ages 65 years and over have an increased risk of IS after Pfizer bivalent booster administration; patients and healthcare providers should not be dissuaded from receiving or administering this booster vaccine.

## Methods

### Data collection, study population, variables, and outcomes

We used the TriNetX platform to access aggregated, de-identified electronic health records (EHR) of over 90 million patients from 56 HCOs across all 50 American states, covering diverse geographic, age, race, and ethnic groups (United States Collaborative Network)^5^. The MetroHealth System, Cleveland Ohio, Institutional Review Board (IRB) has determined that research using the de-identified and aggregated data from TriNetX as described in this study is not Human Subject Research and therefore IRB review was not required. We have previously used the TriNetX platform to study risk factors and outcomes of COVID-19 infection and vaccination^14–16^.

TriNetX data are collected from participating HCOs, primarily from EHR systems comprised of structured demographics, diagnoses, procedures, and medications but also from facts extracted from clinical documents using natural language processing^17^. TriNetX completes intensive data preprocessing to minimize missing values. The platform also maps data to a clinical model with consistent semantic meanings so that the data can be queried consistently regardless of the underlying data source. All covariates are either binary, categorical, or continuous. Missing sex values are represented as “Unknown Sex,” while missing data for race and ethnicity are represented as “Unknown Race” and “Unknown Ethnicity,” respectively. The data available in TriNetX go back decades, depending on the HCO, and are frequently updated (80% of data providers update their data in 1, 2, or 4-week intervals)^18^. For this study, the EHR data were queried and analyzed on October 8, 2023.

**Fig. 2:**
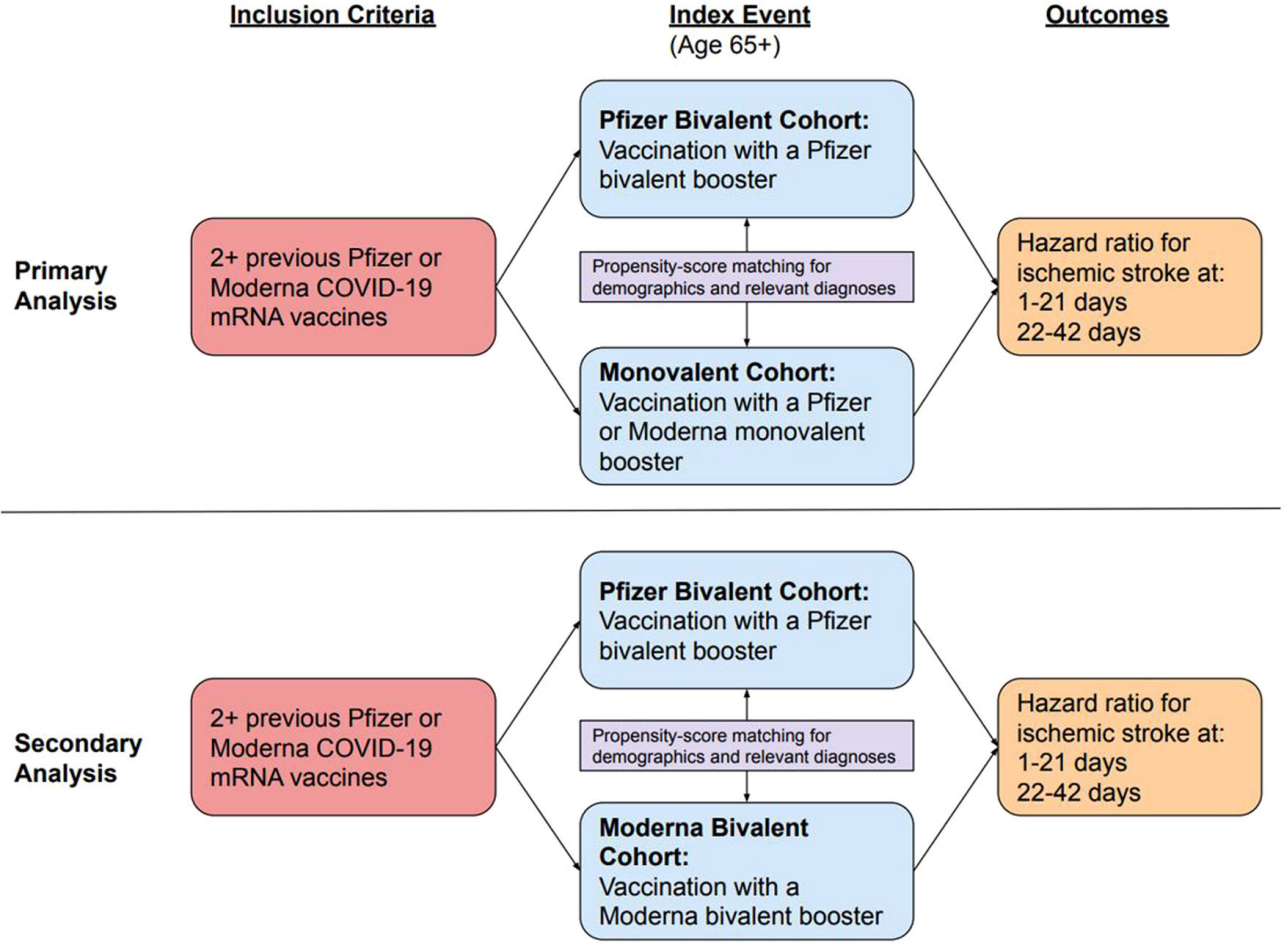
Cohort selection and design.

The primary analysis compared the hazard of IS in patients aged 65 years and over after Pfizer bivalent booster versus monovalent booster; the secondary analysis compared the hazard of IS in patients aged 65 years and over after Pfizer bivalent booster versus Moderna bivalent booster (Fig. 2). The exposure of interest was vaccination by either the Pfizer bivalent booster (“Pfizer bivalent” cohort), Moderna bivalent booster (“Moderna bivalent” cohort), or Pfizer/Moderna monovalent booster (“monovalent” cohort) prior to August 27, 2023, to ensure sufficient time for follow-up at 21 and 42 days (Fig. 2). Patients in the monovalent cohort were included beginning in August 2021, while those in the Pfizer and Moderna bivalent cohorts were included beginning in September 2022, as these time periods represent when the cohorts began receiving booster vaccines in TriNetX. Cohorts were matched by demographics (age, sex, race, ethnicity), COVID-19 infection, medical conditions that are risk factors for both IS and severe COVID-19 infection^19,20^, and adverse socioeconomic determinants of health (Table 1). Self-reported race and ethnicity data in TriNetX come from the clinical EHR systems of the contributing HCOs. TriNetX maps race and ethnicity data from its contributing HCOs to the following categories: (1) Race: Asian, American Indian or Alaskan Native, Black or African American, Native Hawaiian or Other, White, Unknown Race; and (2) Ethnicity: Hispanic or Latino, Not Hispanic or Latino, Unknown Ethnicity. The outcome of interest was an encounter diagnosis for IS in TriNetX at either 1–21 days or 22–42 days after booster administration (Fig. 2). Details of clinical codes for covariates, exposures, and outcomes are described in Supplementary Table 2.

### Statistical analysis

To compare the hazard of IS between the Pfizer bivalent and monovalent cohorts, as well as the Pfizer bivalent and Moderna bivalent cohorts, the cohorts were propensity-score matched (1:1 matching by nearest neighbor greedy matching algorithm with a caliper of 0.25 standard deviations) for the variables enumerated above. Kaplan–Meier survival analysis was used to estimate the probability of IS at 1–21 days or 22–42 days after booster administration. The Kaplan–Meier analysis estimates the probability of an outcome at a respective time interval (daily time interval in this analysis). To account for the patients who exited the cohort during the analysis period, and therefore should not be included in the analysis, censoring was applied. Patients are censored when the last data point in the patient’s record is within the time interval of interest, or if the outcome of interest occurs after the index event but before the start of the time window^21^. The Cox proportional hazard assumption was tested using Schoenfeld residuals^22^. The TriNetX platform calculates HR and associated 95% CI using the R survival package v3.2-3. For generating HR, TriNetX sets robust=FALSE using the R survival package, which is a limitation of the TriNetX platform since it does not consider potential clustering of patients within HCOs or specific geolocations. All statistical tests were conducted in October 2023 within the TriNetX Analytics platform with significance set at *p*-value < 0.05 (two-sided). A sub-analysis was conducted to compare the hazard of first-time IS between the Pfizer bivalent cohort and monovalent cohort, but not between the Pfizer bivalent cohort and Moderna bivalent cohort due to limited sample size.

### Supplementary information

Supplementary Tables

## Data Availability

A subscription to TriNetX Analytics is required to query the aggregated, de-identified patient data analyzed in this study. All relevant data are available from the authors.

## Code availability

The custom code used to generate hazard ratios in this study is proprietary to TriNetX Analytics. A subscription to the TriNetX platform is required for description of this code, which can be found in the TriNetX Help Center once the subscription is obtained.
