# Supplementary material for: Ischemic stroke after COVID-19 bivalent vaccine administration in patients aged 65 years and older in the United States
Source: NPJ Vaccines. 2023 Nov 23;8:180. doi: 10.1038/s41541-023-00777-w (PMC10667491; doi:10.1038/s41541-023-00777-w)
Supplement: Supplementary file 1 — Supplementary Tables [file 41541_2023_777_MOESM1_ESM.pdf]

Supplemental Tables

Supplementary Table 1. Patient characteristics in the Pfizer bivalent cohort and Moderna bivalent cohort before and after propensity-score matching.

Characteristics	Before Matching, %			After Matching, %		
	Pfizer bivalent group (n = 110,667)	Moderna bivalent group (n = 26,962)	Standard mean difference	Pfizer bivalent cohort (n = 26,962)	Moderna bivalent cohort (n = 26,962)	Standard mean difference
**Demographics**						
Age at Index (years, mean±SD)	73.47±6.15	73.87±6.24	0.06	43.11	43.60	0.01
**Sex**						
Male	42.08	43.595	0.03	0.00	0.00	0.01
Female	52.69	55.122	0.05	73.35	72.96	0.01
**Race**						
White	70.89	72.962	0.05	6.20	6.34	0.01
Black or African American	12.18	13.378	0.04	6.42	6.42	0.01
Asian	4.08	6.339	0.10*	0.00	0.00	0.01
Unknown Race	12.09	6.424	0.20*	7.39	7.84	0.00
**Ethnicity**						
Hispanic or Latino	6.75	7.841	0.04	5.31	5.38	0.02
Not Hispanic or Latino	79.13	86.781	0.20*	0.00	0.00	0.02
Unknown Ethnicity	14.13	5.378	0.30*	77.98	78.41	0.00
**Diagnoses**						
Disorders of lipoprotein metabolism and other lipidemias	73.83	78.407	0.11*	32.87	33.17	0.01
Essential (primary) hypertension	70.93	74.913	0.09	34.36	34.72	0.00
Type 2 diabetes mellitus	32.05	33.173	0.02	28.42	29.00	0.01
Overweight and obesity	32.42	34.716	0.05	14.74	15.94	0.01
Ischemic heart diseases	28.05	28.996	0.02	15.91	17.10	0.01
Mental and behavioral disorders due to psychoactive substance use	16.73	15.941	0.02	10.95	11.28	0.03
Cerebrovascular diseases	17.03	17.102	0.00	5.78	6.42	0.03
Nicotine dependence	11.95	11.279	0.02	13.14	13.89	0.01
Cerebral infarction	6.28	6.424	0.01	13.00	13.70	0.03
Atrial fibrillation and flutter	13.62	13.89	0.01	3.74	4.41	0.02
COVID-19	13.86	13.697	0.00	5.76	6.55	0.02
Alcohol related disorders	4.50	4.414	0.00	43.11	43.60	0.03
**Persons with potential health hazards related to socioeconomic and psychosocial circumstances**	7.10	6.55	0.02	55.60	55.12	0.03

*Standard mean difference greater than 0.1, a threshold indicating imbalance.

Supplementary Table 2. Codes for covariates, exposures, and outcomes used in TriNetX.

Covariate	TriNetX Code
**Demographics**	**Code System Concept**
White	2106-3
Black or African American	2054-5
Asian	2028-9
Unknown Race	2131-1
Hispanic or Latino	2135-2
Not Hispanic or Latino	2186-5
**Diagnoses**	**International Classification of Diseases, 10th edition**
Type II diabetes mellitus	E11
Overweight and obesity	E66
Disorders of lipoprotein metabolism and other lipidemias	E78
Mental and behavioral disorders due to psychoactive substance use	F10-F19
Alcohol related disorders	F10
Nicotine dependence	F17
Essential hypertension	I10
Ischemic heart disease	I20-I25
Atrial fibrillation	I48
Cerebrovascular diseases	I60-I69
Cerebral infarction	I63
Persons with potential health hazards related to socioeconomic and psychosocial circumstances	Z55-Z65
COVID-19	U07.1
Ischemic stroke	I63
**Vaccination**	**Current Procedural Terminology**
Pfizer bivalent	91312, 0124A
Moderna bivalent	91313, 0134A
Pfizer monovalent	91300
Moderna monovalent	91301
